# Relation extraction for biological pathway construction using node2vec

**DOI:** 10.1186/s12859-018-2200-8

**Published:** 2018-06-13

**Authors:** Munui Kim, Seung Han Baek, Min Song

**Affiliations:** Department of Library and Information Science, Yonsei University, 50, Yonsei-ro, Seodaemun-gu, Seoul, Republic of Korea

**Keywords:** Biological pathways, Relation extraction, Pathway extraction

## Abstract

**Background:**

Systems biology is an important field for understanding whole biological mechanisms composed of interactions between biological components. One approach for understanding complex and diverse mechanisms is to analyze biological pathways. However, because these pathways consist of important interactions and information on these interactions is disseminated in a large number of biomedical reports, text-mining techniques are essential for extracting these relationships automatically.

**Results:**

In this study, we applied node2vec, an algorithmic framework for feature learning in networks, for relationship extraction. To this end, we extracted genes from paper abstracts using pkde4j, a text-mining tool for detecting entities and relationships. Using the extracted genes, a co-occurrence network was constructed and node2vec was used with the network to generate a latent representation. To demonstrate the efficacy of node2vec in extracting relationships between genes, performance was evaluated for gene-gene interactions involved in a type 2 diabetes pathway. Moreover, we compared the results of node2vec to those of baseline methods such as co-occurrence and DeepWalk.

**Conclusions:**

Node2vec outperformed existing methods in detecting relationships in the type 2 diabetes pathway, demonstrating that this method is appropriate for capturing the relatedness between pairs of biological entities involved in biological pathways. The results demonstrated that node2vec is useful for automatic pathway construction.

## Background

In the field of biology, biological pathway analysis is important for gaining insight into the underlying phenomenon of complex interactions between biological components [1–3]. Biological pathways are constructed based on collective interpretations of biomedical knowledge determined in many different studies, which demands considerable human effort [4]. Specifically, to construct pathways, biologists must read and interpret a large number of biomedical reports [5]. However, with the exponential growth in research papers in biology, it has become increasingly difficult to remain updated on new developments [6, 7], increasing interest in text mining techniques that can detect and extract biological entities, such as gene, disease, and cell and relationships between these entities [8].

Numerous text mining techniques for relationship extraction have been proposed, ranging from a simple but flexible method such as co-occurrence-based relationship extraction [9, 10] to complex techniques including rule-based [11–15], unsupervised [16, 17], and supervised methods [18–24]. However, most studies of relationship extraction have used supervised methods which are feature-based. Feature-based techniques for relationship extraction require a large amount of manually labeled data [17, 25], which is costly and time-consuming. Moreover, feature engineering and extraction are important tasks because the performance of supervised learning techniques is largely dependent on the features [21] and thus requires domain expert knowledge.

To tackle the training data issue, distantly/weakly supervised learning methods have been introduced [26–28]. Specifically, in the distantly supervised approach, an existing knowledge base is used to automatically label entities in the text and annotated data is utilized for training a classifier [29]. Moreover, weakly supervised learning techniques can work with small, inexact, and inaccurate training data [30]. However, these supervised learning techniques depend on the knowledge base in a given scientific domain and labeled data.

Self-supervised learning is a type of supervised learning used for learning representations entirely from unlabeled data such as autoencoders [31], Word2Vec [32], and node2vec [33]. Without training data, we can use these methods for prediction tasks. To take advantage of this strength, in the study, we applied node2vec, a network-embedding algorithm, for relation extraction in biological pathways. Another reason to use node2vec is that relationship extraction can be used as a link prediction between two biological entities in the network. Node2vec can learn the continuous feature representations of nodes in networks by using a biased random walk to sample neighborhoods of nodes [33]. As such, without annotated data, node2vec can learn rich feature representations for all nodes in a network.

As a result, in this study, we predicted whether two biological entities can be connected in a network using the node2vec algorithm. A series of experiments showed that the network embedding technique is well-suited for relationship extraction between genes in a biological pathway.

## Results

### Evaluation of gene-gene interactions in the type 2 diabetes pathway

**Fig. 1:**
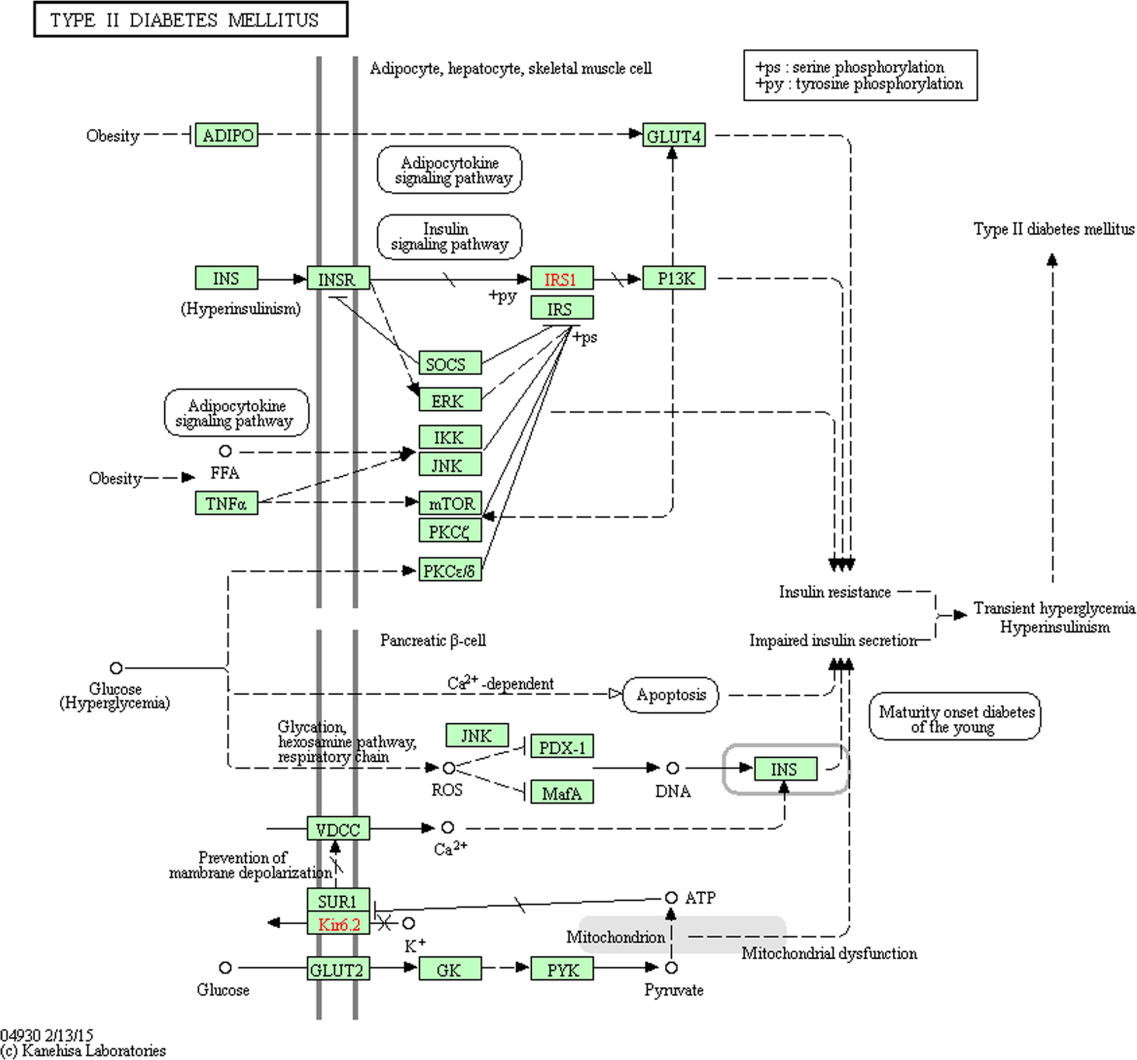
Type 2 diabetes pathway. Pathway data ©2017 KEGG. Retrieved December 24, 2017, from http://www.genome.jp/kegg-bin/show_pathway?hsa04930. Screenshot by author

The type 2 diabetes mellitus pathway consists of 25 genes, 14 other biological components, such as disease and molecular function, and their direct/indirect relationships. Figure 1 shows the type 2 diabetes pathway provided by the KEGG PATHWAY database [34]. It is well-known that type 2 diabetes is strongly associated with insulin resistance [35]. Therefore, we focused on the pathways related to ‘insulin resistance’ within the type 2 diabetes pathway of KEGG. Specifically, the pathways linked to ‘insulin resistance’ contained 19 biological entities, including gene, molecular function and disease, and 26 connections between these entities, as shown in Fig. 1. These biological components are listed in Table 1.

**Table 1:** Entities belonging to the paths connected to insulin resistance

No.	Entity	Type	No.	Entity	Type
1	ADIPO	Gene	11	IKK	Gene
2	GLUT4	Gene	12	JNK	Gene
3	INS	Gene	13	mTOR	Gene
4	INSR	Gene	14	PRKCZ	Gene
5	IRS1	Gene	15	PKCD/E	Gene
6	IRS	Gene	16	Obesity	Disease
7	PI3K	Gene	17	Transient hyperglycemia	Disease
8	SOCS	Gene	18	Type 2 diabetes mellitus	Disease
9	ERK	Gene	19	FFA	Molecular Function
10	TNF-alpha	Gene			

**Fig. 2:**
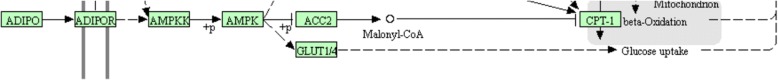
Interaction between adiponectin and GLUT4 in the adipocytokine signaling pathway. Pathway data ©2017 KEGG. Retrieved December 24, 2017, from http://www.genome.jp/kegg-bin/show_pathway?hsa04920. Screenshot by author

Among the 25 relationships, we evaluated 18 gene-gene interactions in the type 2 diabetes pathway where the relationships between genes are made up of the most part. The 18 pairs of genes and relationship types (direct/undirect) are shown in Table 2. The interactions shown in the KEGG type 2 diabetes pathway fall into 2 categories: direct and indirect interactions. The dotted lines in the KEGG pathway denote an indirect relationship between two biological entities. Two entities in an indirect relationship interact each other though several other entities. Specifically, as shown in Table 2, the relationship between ADIPO and GLUT4 is indirect because the two genes are engaged in the adipocytokine signaling pathway as well as the type 2 diabetes pathway, as the biological components can participate in multiple pathways. Figure 2 shows that these two genes interact with each other through ADIPOR, AMPKK, and AMPK.

**Table 2:** Eighteen gene-gene interactions and, interaction type, and another participating pathway of each relationship

Relation no.	Starting entity	Target entity	Interaction type	Another participating pathway
1	ADIPO	GLUT4	Indirect	Adipocytokine signaling pathway
2	INS	INSR	Direct	–
3	INSR	IRS1/IRS	Direct	–
4	IRS1/ IRS	PI3K	Direct	–
5	INSR	SOCS	Direct	–
6	SOCS	IRS1/IRS	Direct	–
7	INSR	ERK	Indirect	Insulin signaling pathway
8	ERK	IRS1/IRS	Indirect	Insulin signaling pathway
9	PI3K	GLUT4	Indirect	Insulin signaling pathway
10	PI3K	mTOR	Indirect	Insulin signaling pathway
11	PI3K	PKC	Indirect	Insulin signaling pathway
12	TNFA	IKK	Indirect	Adipocytokine signaling pathway
13	TNFA	JNK	Indirect	Adipocytokine signaling pathway
14	TNFA	mTOR	Indirect	Adipocytokine signaling pathway
15	IKK	IRS1/IRS	Direct	–
16	JNK	IRS1/IRS	Direct	–
17	PKCZ	IRS1/IRS	Direct	–
18	PKCD/E	IRS1/IRS	Direct	–

Accordingly, we expanded indirect interactions involved in the type 2 diabetes pathway, if two entities in a given indirect relationship participate in another pathway, to capture more detailed information on the process of interactions. Among the 18 gene-gene interactions, 9 were found to be indirect and pathway information in which each pair of genes participate in the type 2 diabetes pathway is described in Table 2.

Therefore, these 9 indirect paths were expanded to identify direct relationships. Table 3 reports the extended paths and direct paths in each extended path. For PI3K and GLUT4 (relationship no. 9), there are three possible ways to connect from PI3K to GLUT4: PI3K-PKC-GLUT4, PI3K-PDK1/2-PKC-GLUT4, and P13K-PDK1/2-AKT-GLUT4. Because PKC, the upstream kinase, is responsible for the phosphorylation and activation of AGC kinase members regulated by PI3K [36–42], we selected the two expanded paths, P13K-PDK1/2-AKT-GLUT4 and P13K-PDK1/2-PKC-GLUT4. As a result, including the 9 direct links in the type 2 diabetes pathway (relationship no. 2, 3, 4, 5, 6, 15, 16, 17, and 18), a total of 30 direct gene-gene interactions were used for performance evaluation.

**Table 3:** Extended paths and directed links in each expanded path

Relation no.	Extended path based on the KEGG pathways	Direct relationship no.	Direct paths in the expanded path
1	ADIPO-ADIPOR1-AMPK-GLUT4	1	ADIPO-ADIPOR
		2	AMPKK- AMPK
7 8	INSR-SHC-GRB2-SOS-Ras-Raf-MEK1/2-ERK1/2 IRS-GRB2-SOS-Ras-Raf-MEK1/2-ERK1/2	3	INSR-SHC
		4	SHC-GRB2
		5	GRB2-SOS
		6	SOS-Ras
		7	Ras-Raf
		8	Raf-MEK1/2
		9	MEK1/2-ERK1/2
		10	IRS-GRB2
9 10 11	PI3K-PDK1/2-AKT-GLUT4 PI3K-PDK1/2-PKC-GLUT4 PI3K-PDK1/2-AKT-mTOR PI3K-PDK1/2-PKC	11	PI3K-PDK1/2
		12	PDK1/2-AKT
		13	AKT-GLUT4
		14	PDK1/2-PKC
		15	PKC-GLUT4
		16	AKT-mTOR
12 13 14	TNFA-TNFR1-TRADD-TRAF2-IKK TNFA- TNFR1-TRADD-TRAF2 -JNK TNFA-TNFR1-TRADD-TRAF2-mTOR	17	TNFA-TNFR1
		18	TNFA-TNFR2
		19	TNFR1-TRADD
		20	TRADD-TNFR2
		21	TNFR2-TRAF2

Each entity participating in the direct interactions was selected in sequence as a starting node to discover its closest terms. With each starting node, the 100 most similar genes were extracted by calculating cosine similarity between a given starting gene and other gene vectors. Next, the starting gene was paired with each of the 100 extracted genes and the newly generated relationships were ranked by cosine similarity. We evaluated the performance of node2vec by examining whether a given path in the type 2 diabetes pathway was ranked high in the results. For instance, when INS was a starting node, genes with high similarity to INS were extracted and the ranking of INSR was examined. If a direct path was not shown in the 100 newly created relationships, we considered that node2vec did not capture the path.

**Fig. 3:**
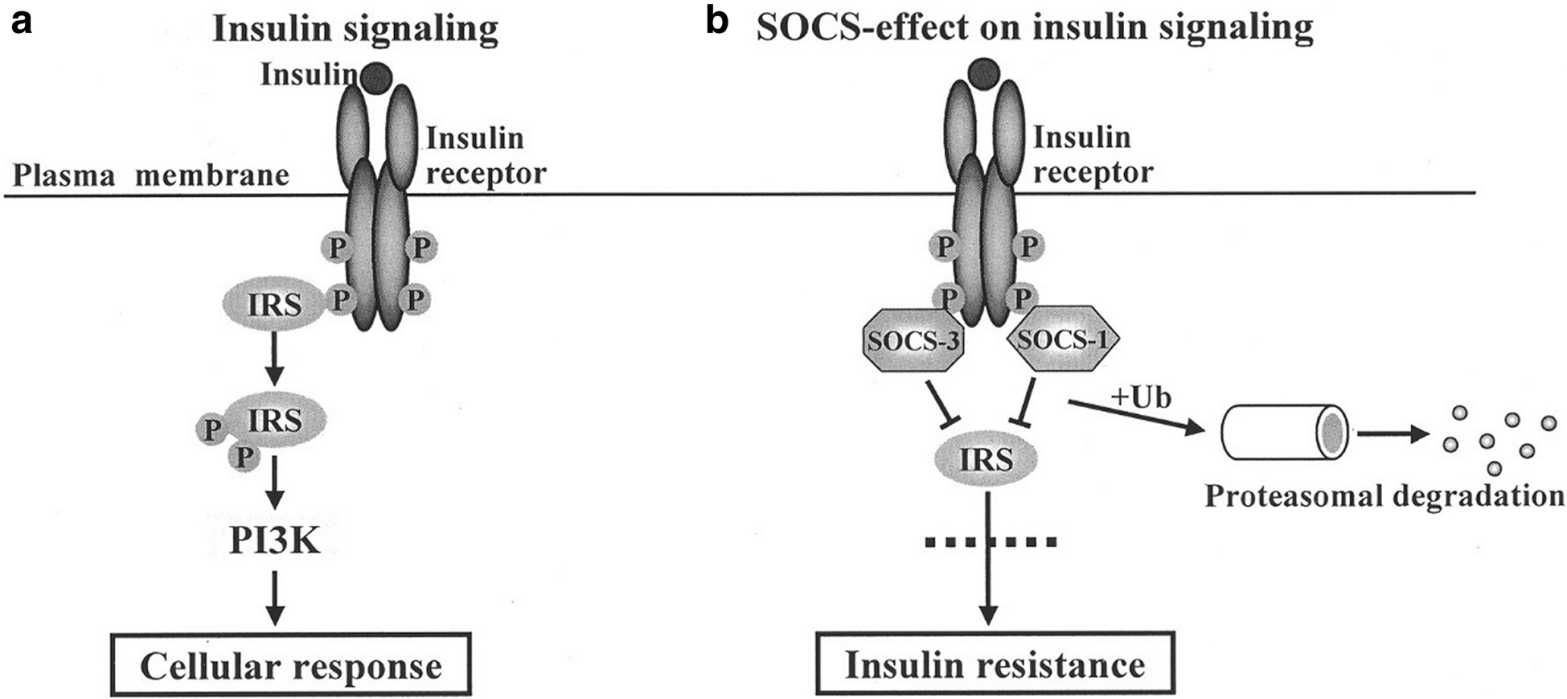
INS-INSR and IRS-PI3K interactions in insulin signaling (**a**), and the effect of SOCS on insulin signaling (**b**). Adapted from “Diabetes and suppressors of cytokine signaling proteins,” by S. G. Rønn, N. Billestrup and T. Mandrup-Poulsen, 2007, Diabetes, 56(2), 541–548, p

The 30 direct links and ranking of each relationship are described in Table 4. Specifically, among these 30 links, 25 gene-gene interactions were ranked within 100. Moreover, the 24 direct relationships were ranked within 10 (direct relationship no. 1–11, 13, 16–27). For example, IRS1/IRS-PI3K, INSR-SOCS, and SOCS-IRS1/IRS (direct relationship no. 3, 4, and 5) directly interact with each other in the type 2 diabetes pathway, which is supported by the additional pathway information shown in Fig. 3.

**Table 4:** Thirty direct gene-gene interactions and the ranking of each link

Direct relation no.	Starting entity	Target entity	Ranking (node2vec)	Ranking (co-occurrence)	Ranking (DeepWalk)
1	INS	INSR	10/100	–	–
2	INSR	IRS1/IRS	4/100	1/100	7/100
3	IRS1/ IRS	PI3K	1/100	3/100	1/100
4	INSR	SOCS	8/100	10/100	6/100
5	SOCS	IRS1/IRS	2/100	3/100	2/100
6	IKK	IRS1/IRS	2/100	2/100	2/100
7	JNK	IRS1/IRS	4/100	5/100	4/100
8	PKCZ	IRS1/IRS	6/100	–	7/100
9	PKCD/E	IRS1/IRS	2/100	4/100	2/100
10	ADIPO	ADIPOR	1/100	1/100	1/100
11	AMPKK	AMPK	1/100	1/100	1/100
12	INSR	SHC	38/100	–	67/100
13	SHC	GRB2	5/100	5/100	16/100
14	GRB2	SOS	–	–	–
15	SOS	Ras	–	–	–
16	Ras	Raf	4/100	6/100	–
17	Raf	MEK1/2	1/100	5/100	4/100
18	MEK1/2	ERK1/2	1/100	1/100	1/100
19	IRS1/IRS	GRB2	4/100	4/100	4/100
20	PI3K	PDK1/2	1/100	2/100	6/100
21	PDK1/2	AKT	3/100	4/100	5/100
22	AKT	GLUT4	4/100	4/100	4/100
23	PDK1/2	PKCZ	1/100	1/100	1/100
24	PKCZ	GLUT4	3/100	2/100	3/100
25	AKT	mTOR	1/100	1/100	1/100
26	TNFA	TNFR1	6/100	1/100	6/100
27	TNFA	TNFR2	4/100	1/100	6/100
28	TNFR1	TRADD	–	–	–
29	TRADD	TNFR2	–	–	–
30	TNFR2	TRAF2	–	–	–
Total number of links captured by node2vec, co-occurrence and DeepWalk	25	22	23

According to a previous study [43], insulin (INS) binding to the insulin receptor (INSR) initiates phosphorylation of the receptor and IRS proteins, which activates PI3K. Moreover, SOCS interacts with the phosphorylated receptor, preventing the binding and activation of IRS proteins.

The results listed in Table 4 show that INS is a close term to INSR (ranking 10th), INSR to IRS1/IRS (ranking 4th), IRS1/IRS to PI3K (ranking first), INSR to SOCS (ranking 8th), and SOCS to IRS1/IRS (ranking second). These results indicate that node2vec can accurately reflect the relatedness of two directly related genes, demonstrating the possibility to applying node2vec for relationship extraction.

However, the ranking of the 5 direct paths is not included in the top 100. These results show that node2vec cannot capture the similarity between two entities belonging to these paths because biological entities and relationships among them were not observed in type 2 diabetes–related papers. This issue will be further described in the Discussion section.

Moreover, we compared our results with those generated by the baseline methods, co-occurrence and DeepWalk. To compare the 3 different techniques, node2vec, co-occurrence, and DeepWalk, we extracted 100 co-occurring gene pairs as well as DeepWalk-generated pairs with every starting node of the 30 direct links. First, co-occurring links were ranked by their co-occurrence counts. For example, in the case of direct link no. 1, genes frequently co-occurring with INS were extracted such as INS-GLP-1(co-occurrence frequency: 3959) and INS-TNF-alpha (co-occurrence frequency: 3145). The co-occurrence link, INS-INSR (co-occurrence frequency: 1819), was ranked 9th. Moreover, DeepWalk-generated gene paths were ranked as paths generated by node2vec.

The results are listed in Table 4. Specifically, 22 co-occurring links and 25 paths generated using node2vec were ranked within the top 100. Thus, node2vec reflects the relatedness of two genes belonging to the 3 paths (direct relationships no. 1, 8, and 12) better than co-occurrence. These 3 links were not observed in the co-occurrence results. For the ranking of the 30 direct relationships, only 4 co-occurring path rankings were higher than those of the 4 links generated using node2vec (direct relationships no. 2, 24, 26, and 27). In contrast, 9 node2vec-generated paths (direct relationships no. 3, 4, 5, 7, 9, 16, 17, 20, and 21) were ranked higher than the co-occurrence links. The remaining 9 path rankings were identical.

In addition, 23 DeepWalk-generated paths are ranked within the top 100, revealing that 2 additional direct paths (direct relationships no. 1 and 16) were captured by node2vec. For the ranking of the 30 direct relationships, only 1 DeepWalk path (direct relationship no. 4) ranked higher than the node2vec-generated path. In contrast, the rankings of 8 paths generated by node2vec were higher than those by DeepWalk. The remaining 14 paths showed the same rank. These results demonstrate that node2vec performs better than co-occurrence and DeepWalk in capturing the relatedness of two genes in the extended type 2 diabetes pathway.

## Discussion

In the study, we applied the node2vec algorithm to extract direct paths in a biological pathway. The results revealed the possibility of its application in automated pathway extraction. We further examined if node2vec can capture the directions between pairs of biological components in the pathway. It is essential to extract these directions because biological reactions in the pathway flow from reactants to products, in reverse, or both [44, 45].

The directions were expressed from starting nodes to target nodes, which means that the biological reaction between a given gene pair flows from the starting entity to the target entity. To investigate whether node2vec reflects the directions in the ranking, we changed the position of two entities in the 5 pairs such as INSR-IRS1/IRS, AMPKK-AMPK, Raf-MEK1/2, MEK1/2-ERK1/2, and PKCZ-GLUT4. Next, we set the original target genes (INSR, AMPKK, Raf, MEK1/2, and PKCZ) as starting entities, and the top 100 closest genes were extracted and ranked by similarity between pairs of entities.

The results are presented in Table 5, which shows that the ranking of the newly generated paths were much lower than those of the original links. Specifically, in the case of the direct relationship no. 2 and 24, the newly generated paths are not shown in the results. Thus, the target genes, INSR, AMPKK, Raf, MEK1/2, and PKCZ, were not extracted as similar genes of the starting nodes, IRS1/IRS, AMPK, MEK1/2, ERK1/2, and GLUT4. Based on our results, node2vec can capture the direction of flow between two genes, although an input network and co-occurrence network was not directed.

**Table 5:** Ranking reflecting the reverse directions

Direct relation no.	Original starting node	Original target node	Original ranking	New starting node	New target node	New ranking
2	INSR	IRS1/IRS	3/100	IRS1/IRS	INSR	–
11	AMPKK	AMPK	1/100	AMPK	AMPKK	7/100
17	Raf	MEK1/2	1/100	MEK1/2	Raf	6/100
18	MEK1/2	ERK1/2	1/100	ERK1/2	MEK1/2	55/100
24	PKCZ	GLUT4	3/100	GLUT4	PKCZ	–

**Fig. 4:**
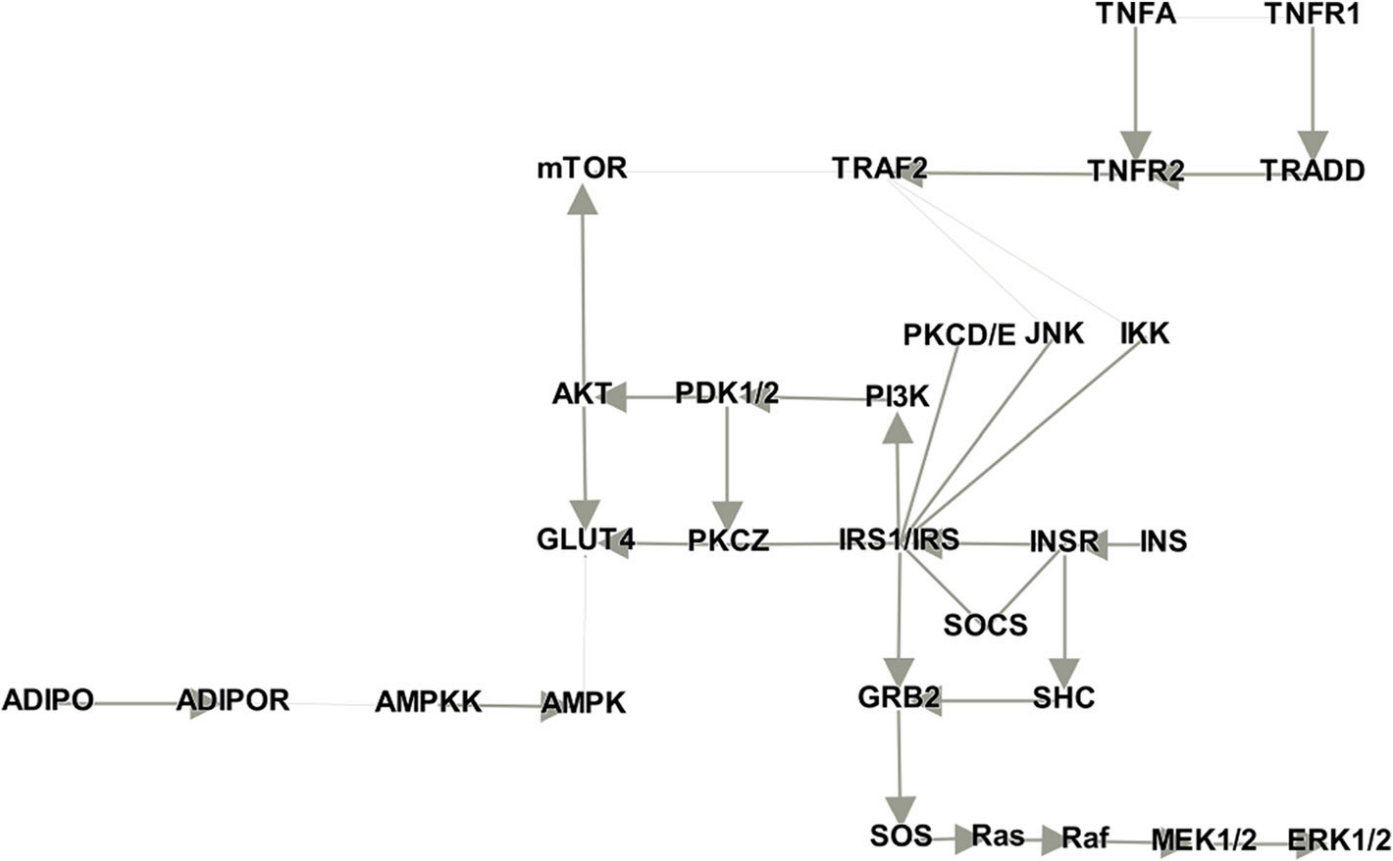
Extended type 2 diabetes pathway network

In addition to the flow directions, in a pathway network, hub nodes exist showing the highest degree [46, 47]. Hub genes are considered important because these genes are likely essential for organism survival [48]. To identify the hub genes and determine how well node2vec captures the relatedness between these hubs, we constructed an extended type 2 diabetes pathway network using other 2 pathways: insulin signaling pathway and adipocytokine signaling pathway. This expanded network was visualized using Gephi [49], a network visualization tool, which is illustrated in Fig. 4.

The extended network consists of 29 genes and 35 edges between these entities. The thickness of the edges represents directed or undirected links among genes and 5 edges in the thin lines are undirected links (mTOR-TRAF2, TRAF2-JNK, TRAF2-IKK, GLUT4-AMPK, and ADIPOR-AMPKK). Nodes with a high degree of centrality indicate hub genes in the extended network. Specifically, IRS1/IRS showed the highest degree centrality (8), demonstrating that IRS1/IRS is a hub gene in the network. Genes connected directly to IRS1/IRS are INSR, PI3K, SOCS, IKK, JNK, PKCZ, PKCD/E, and GRB2. These 8 direct links were ranked within the top 10 in the node2vec results, showing that node2vec is an appropriate technique for extracting important relationships in the network, which is essential for pathway construction.

Moreover, the node2vec model was generated based on the adjacency matrix of biological components extracted from the text. Therefore, if entities and relationships are not extracted from the text and not included in the adjacency matrix, it is less likely that node2vec can capture the relatedness of two entities. For direct relationships such as TNFR1-TRADD, TRADD-TNFR2, TNFR2-TRAF2, GRB2-SOS, and SOS-Ras, similarities between these gene pairs is not captured by node2vec. This is because the number of abstracts including each pair of genes is zero, as shown in Table 6.

**Table 6:** Number of abstracts including direct gene pairs

Entity pair	Number of abstracts
TNFR1-TRADD	0
TRADD-TNFR2	0
TNFR2-TRAF2	0
GRB2-SOS	0
SOS-Ras	0

Specifically, 5 gene pairs in Table 6 are not shown in the abstracts but rather are shown in the full-text. As such, using full-text papers available in PMC would be helpful for constructing a more precise co-occurrence network, which can enhance the performance of node2vec.

## Conclusions

In the study, we applied node2vec to extract relationships between biological entities in the extended type 2 diabetes pathway. We showed that node2vec successfully extracted a high percentage of gene pairs belonging to the expanded pathway. Moreover, it outperformed existing techniques such as co-occurrence and DeepWalk. In addition, we demonstrated that node2vec captured the direction flow between two genes, which is essential because reaction flow exists in biological pathways. Accordingly, it has been shown that node2vec is a suitable technique for extracting relationships between entity pairs in pathways.

However, we evaluated our results based on the existing relationships in the pathway for path prediction and thus the relationships extracted using node2vec that have not been verified should be further examined, which is the main theme of our follow-up study. Moreover, several direct paths detected in our extended pathway were not captured by node2vec. As described in the Discussion section, two entity names belonging to the paths did not appear in abstracts but rather in full-text articles. Accordingly, if we use full-text articles, it will be possible to construct a more exquisite co-occurrence network, which ultimately increases node2vec performance in extracting relationships of biological pathways. This is another principal topic of our follow-up study.

## Methods

To demonstrate the efficacy of node2vec for relation extraction, we selected ‘type 2 diabetes’ as a case study. The methodology used in this study is described below.

### Data collection

Type 2 diabetes-related data were collected from PubMed, which contains over 26 million references to journal articles in life sciences on biomedicine. We used the keyword ‘type 2 diabetes’ to retrieve all papers indexed with this search term. Only articles including the term in the titles and abstracts were collected. PubMed XML records were retrieved using EFetch API [50]. As a result, the total number of collected records was 99,689 papers, published from 1978 to 2018. Finally, PMIDs, titles, and abstracts were extracted from the XML records and preprocessed for entity and relationship extraction.

### Entity and relation extraction

For entity extraction, PKDE4J [14], a biomedical text mining tool, was utilized. Using the tool, biomedical entities can be extracted either by dictionary or supervised learning, or both. In our experiment, a combination approach was used to extract biological entities. Specifically, candidates of the biological entities were identified using the Stanford NER model [51] and the candidates were mapped into the Unified Medical Language System (UMLS) concepts to decrease false-positives. The UMLS is a vocabulary database of biomedical concepts and relationships among concepts, developed by the National Library of Medicine. The biomedical concepts in the UMLS Metathesaurus are categorized into 143 semantic types [52]. As such, semantic types can be selected to extract specific types of entities. In this study, semantic types matching Gene/Protein were used for gene extraction from biomedical text. These entity types are Cell component, Gene or Genome, Enzyme, Receptor, Nucleic acid, Nucleoside, or Nucleotide, Amino acid, Peptide or Protein, Molecular sequence, Nucleotide sequence, and Amino acid sequence.

For relationship extraction, two biological components were linked when the entities were mentioned together in the same sentence. The assumption behind this approach is that frequently co-occurring entities in the same sentence are more likely to be related than those occurring together in the same abstract. A co-occurrence network in which nodes and edges represent biological entities and co-occurrence relationships, respectively, was constructed and used as an input for node2vec.

### Node2vec for latent path prediction

Node2vec is “an algorithmic framework for learning continuous feature representations for nodes in the networks” [33]. It can be used for path prediction in the network by maximizing the probability of preserving network neighborhoods of nodes via second order random walk [33]. In the networks, nodes exhibit homophily, structural equivalence, or both. Thus, node2vec employs biased random walks in which return parameter p and in-out parameter q adjust the walks to sample neighborhood of nodes that lead to embeddings corresponding to the structural, homophily equivalence, or both. Node2vec improves the random walk phase of DeepWalk [53], another feature learning technique for networks, by introducing hyperparameters that control the depth and breadth of random walks. Many studies have shown that node2vec outperforms DeepWalk [54–56].

Specifically, in the random walks process, if the return parameter is high (>max(q, 1)), the walk is less inclined to visit already visited nodes. In contrast, if p is low (<min(q,1)), the search is restricted to nearby nodes, which is essential for ascertaining structure equivalence [33]. For the in-out parameter q, if q is less than 1 (q < 1), we are more likely to sample nodes that are further away from a source node. Thus, “the sampled nodes more accurately reflect a micro-view of the neighborhood [33]”, which is crucial for discovering communities/clusters on homophily. The goal of our study was to identify nodes that are closely interconnected and belong to the same communities (homophily equivalence [33, 57]), and we set *p* = 1 and q = 0.5.

In addition to the p and q parameters, we set other parameters involved in node2vec as d = 128, r = l = 10, and k = 10 where d, r, l, and k denote embedding dimensions, walk per node, walk length, and context size, respectively. Parameter values were selected based on the parameter-sensitive part of the original paper [33] for the best performance. Moreover, to accurately compare node2vec with DeepWalk, we used the same parameters for both methods.

The constructed co-occurrence network was used as input for node2vec and DeepWalk to learn rich feature representation for every node in the network. Node2vec extends the Skip-gram architecture [58] to networks, learns node embeddings by generating random walks and optimizes the network-based objective function using SGD.

With the embedding matrix, the relatedness between each pair of biological entities (e1, e2) shown in the biological pathway, was identified by computing the cosine similarity of their corresponding transformed vectors (v_e1_, v_e2_).

### Performance evaluation

To evaluate node2vec performance for predicting relationships between biological components, pathway-based analysis was conducted. Specifically, the type 2 diabetes mellitus pathway sourced from the KEGG PATHWAY database was used for the evaluation task. The pathway map in the KEGG PATHWAY provides knowledge regarding diverse molecular networks composed of nodes such as orthologs, genes, small molecules, and their reactions and interactions [34]. As such, node2vec performance was evaluated based on the entity-entity relationships shown in the KEGG pathway map. Moreover, we compared node2vec results with those generated by other baseline methods such as co-occurrence and DeepWalk.

## Abbreviation

UMLS: Unified Medical Language System
